# 4195 “Learning Shots” are an Innovative, Versatile Educational Tool for Clinical and Translational Science

**DOI:** 10.1017/cts.2020.197

**Published:** 2020-07-29

**Authors:** Jean Eby, Susie Hoffman, Karen Johnston, Jennifer Phillips

**Affiliations:** 1 University of Virginia

## Abstract

OBJECTIVES/GOALS: To demonstrate how brief online audiovisual presentations, “learning shots”, informed by evaluation, can be used to quickly and effectively provide essential just-in-time research-related education in the complex and evolving world of clinical and translational science. METHODS/STUDY POPULATION: “Learning shots” are an educational tool, originally developed by the University of Virginia IRB for Health Sciences Research, that cover a broad spectrum of methodological, regulatory, and ethical topics in research. They are designed to be responsive to adult learners, a rapidly changing research environment, and the need for flexible offerings. Learning shots target different groups involved in research including clinical research coordinators, investigators, and trainees. A survey was used to assess the role of learning shots in meeting learning needs. Moving forward, continuous evaluation will occur through the addition of tracking and a short evaluation survey to each learning shot. RESULTS/ANTICIPATED RESULTS: The University of Virginia has an online library of over 30 learning shots. Learning shots are used to cover foundational topics (e.g. GCP) as well as more specialized topics (e.g. comparative effectiveness research). They can also be used to quickly respond to breaking issues (e.g. single IRB review). In an initial survey targeting University of Virginia clinical research coordinators, 54 (68%) of 79 respondents reported having viewed a learning shot. Among those who had, 41 (84%) of 49 respondents reported that learning shots were helpful to their learning needs. Continuous evaluation is expected to further inform how learning shots meet clinical and translational science education needs. DISCUSSION/SIGNIFICANCE OF IMPACT: Learning shots are an innovative and versatile educational tool for clinical and translational science that can be used to quickly and effectively convey important research information in response to an increasingly complex research environment and diverse learner needs.

